# Diagnosis, Manifestations, Laboratory Investigations, and Prognosis in Pediatric and Adult Cushing’s Disease in a Large Center in China

**DOI:** 10.3389/fendo.2021.749246

**Published:** 2021-11-19

**Authors:** Xueqing Zheng, He Wang, Wentai Zhang, Shanshan Feng, Yifan Liu, Shuo Li, Xinjie Bao, Lin Lu, Huijuan Zhu, Ming Feng, Renzhi Wang

**Affiliations:** ^1^ Department of Neurosurgery, Pituitary Centre, Peking Union Medical College Hospital, Peking Union Medical College and Chinese Academy of Medical Sciences, Beijing, China; ^2^ Peking Union Medical College, Chinese Academy of Medical Sciences, Beijing, China; ^3^ Department of Endocrinology, Peking Union Medical College Hospital, Peking Union Medical College and Chinese Academy of Medical Sciences, Beijing, China

**Keywords:** Cushing’s disease, adrenocorticotrophin hormone, cortisol, pediatric, transsphenoidal pituitary surgery

## Abstract

**Purpose:**

Cushing’s disease (CD) is a rare disease that contributes to 70–80% hypercortisolemia, which presents similarities and differences between pediatric and adult patients, and even between male and female patients. However, the comparative study of CD between different age groups and different genders is still insufficient. The aim of the study is to make a systematic comparison to reveal the gender differences in children and adult patients of CD, helping clinicians to provide optimal treatment for different groups of patients.

**Methods:**

We conducted a retrospective research consisting of 30 pediatric and 392 adult CD patients in a single center in Peking Union Medical College Hospital. All 422 patients showed symptoms related to hypercortisolism and received adenoma excision surgery in the department of neurosurgery between 2014 and 2020.

**Results:**

For the accuracy of diagnosis, the sensitivity of BIPSS at baseline in pediatric patients was lower than in adults (75 *vs.* 91%, P = 0.054) but increased greatly after desmopressin stimulation (94 *vs.* 95%). However, the accuracy of lateralization for BIPSS was not preferred for prediction. As for clinical manifestations, growth retardation, weight gain, hirsutism, and acne were more prevalent for children, while for adults, hypertension, osteopenia, glucometabolic disorder, easy bruising, hair loss, and weight loss were more frequently seen. As previously reported, we observed a significant difference between the male prevalence of pediatric and adult patients (50 *vs.* 17%, P < 0.001), which was possibly caused by the more severe and earlier onset of a series of symptoms. Gender-related comparison showed greater morbidity of nephrolithiasis, hypokalemia, hypertension, easy bruising, osteopenia, and striae for male patients, while irregular menses, hirsutism, and hair loss were more common for female patients. Further analysis showed that the secretory activity of the PA axis was higher for males, presenting as the more remarkable alteration of laboratory parameters and contributing to the more severe clinical manifestations. For patients treated with transsphenoidal pituitary surgery (TSS), the immediate prognosis could be predicted by operation history, invasiveness, Ki-67, and information provided by MRI, including tumor size and Knosp grading. However, we still lack methods to predict long-term prognosis.

**Conclusions:**

Our study is the first detailed and systematic comparison between pediatric and adult CD patients. Further exploration of the impact of CD on different genders reveals a more severe and probably an earlier-onset pattern of CD for male patients.

## 1 Introduction

Cushing’s disease (CD) is a rare disease characterized by hypercortisolemia, caused by the excess secretion of adrenocorticotrophin hormone (ACTH) by a pituitary adenoma that stimulates overproduction of cortisol from the adrenal glands (1). Since hypercortisolemia can lead to a series of metabolic disorders and profoundly impact the quality of life, the accurate diagnosis and effective treatment can be very challenging yet critical. Compared with adults, CD is extremely rare in the pediatric population and results in multisystem disorder, including growth retardation, delayed sexual development, obesity, hypertension, glucose intolerance, and mood changes (2). Transsphenoidal pituitary surgery (TSS) is considered as the first-line treatment for CD, but the difficulty, effectiveness, and prognosis of TSS for children may be different from adult patients (3). However, detailed and systematic comparisons of diagnostic accuracy, clinical manifestations, laboratory investigations, and prognosis between pediatric and adult patients have seldom been performed. Previous studies have shown that different from female preponderance in adult patients, the proportion of males was much higher in pediatric patients, but the reason is still yet to know (4). Therefore, it is very meaningful to reveal the gender differences in children and adult populations on symptoms and laboratory parameters of CD, helping clinicians to accurately diagnose and provide optimal treatment for different groups of patients.

To study the impact of CD on different age groups and different genders, we present a retrospective research consisting of 30 pediatric and 392 adult CD patients in a single center who received pituitary adenoma excision operation by an experienced surgeon. After collecting the clinical data, we described and compared the diagnosis, clinical manifestation, laboratory investigations, and prognosis between children- and adult-onset CD, and further analyzed the gender-related differences in different age groups.

## 2 Materials and Methods

### 2.1 Patients

The study population comprised 30 pediatric patients (age <18; 15 males and 15 females; mean age 13.5 ± 3.7 years, range 5–17 years) and 392 adult patients (66 males and 326 females; mean age 39.6 ± 11.5 years, range 18–73 years) (**Table 1**). All 422 patients showed symptoms related to hypercortisolism and were treated in the department of neurosurgery in Peking Union Medical College Hospital (Beijing, China) between 2014 and 2020. All patients received pituitary adenoma excision operation by an experienced surgeon, MF. Among them, 65 adults and 4 children had received surgery before, 1 adult had only received radiotherapy before, and the other 326 adults and 26 children were classified as patients with the first-time operation.

**Table 1:** Basic information of pediatric and adult-onset CD.

	Pediatric CD patients (n = 30)	Adult CD patients (n = 392)	*P* value
**Gender (Female:Male)**	15:15	326:66	**<0.001**
**Age at surgery**	15.0 (17.0–11.5)	38.5 (48.0–30.0)	
**Age at symptom onset**	12.0 (14.3–9.0)	33.0 (43.0–26.0)	
**Course**	1.7 (3.0–1.2)	3.0 (7.0–1.8)	**0.005**
**First time operation (%)**	26 (87)	326 (83)	0.80
**TSS surgery (%)**	30 (100)	387 (99)	1
**Microadenomas on MRI (%)**	14 (74)	237 (81)	0.55
**Invasiveness (%)**	2 (7)	23 (6)	0.70
**CSF leakage (%)**	0 (0)	16 (4)	0.62
**Immediate remission (%)**	24 (80)	293 (76)	0.60
**Long-term remission (%)**	22 (79)	274 (79)	0.99
**Recurrence (%)**	3 (11)^a^	22 (6)	0.42

^a^The three recurrent pediatric CD patients were all male.

The bolded values showed the significant results (P < 0.05). No other significant meanings were related

### 2.2 Inclusion Criteria

Patients were included in this study if they met the standards below:

(1) In immunohistochemical staining, excised tissue in surgery for 87% patients was confirmed as pituitary adenoma in histopathological analysis by ACTH(+).
(2) For 13% patients, the histopathological analysis failed to confirm the excised tissue as pituitary adenoma, which was possible due to the small size of pituitary adenoma. We consider the combination result of patients’ clinical manifestations and auxiliary examinations. As mentioned above, all patients presented clinical manifestations of hypercortisolemia. Laboratory examinations include 24-h urinary free cortisol (UFC) measurement, 8 a.m. F (serum cortisol) and 8 a.m. ACTH, low-dose and high-dose dexamethasone suppression tests (LDDST and HDDST), bilateral petrosal sinus sampling (BIPSS) with desmopressin stimulation. Magnetic resonance imaging (MRI) scan also provided substantial evidence as an imaging examination.

### 2.3 Auxiliary Examinations of Cushing’s Disease

For etiologic diagnosis, an increased level of 24-h UFC indicates hypercortisolism (>103.5 μg/d), which is further supported by increased 8 a.m. F (>22.3 μg/dl) and 8 a.m. ACTH (>46 μg/dl). Pseudo-Cushing’s syndrome can be excluded by LDDST if 24-h UFC on the second day is suppressed below 12.3 μg/d. For localization diagnosis, suppression of 24-h UFC under 50% after HDDST test acts as strong evidence of ACTH-secreting pituitary adenoma. Chest and abdomen CT excluded patients with adrenal tumors. Preoperative pituitary MRI and BIPSS were performed for localization diagnosis. MRI with incomplete data or negative results were defined as inconclusive. MRI also provides information on the size and invasiveness (Knosp ≥3). Due to the high cost and limited availability of CRH in China, we used BIPSS with desmopressin stimulation to indicate pituitary ACTH secretion if central to peripheral ACTH ratio ≥2 pre- or ≥3 post-stimulation. Lateralization is predicted if the inter-petrosal sinus gradient ≥1.4. For lateralization analysis, we excluded patients with negative or incomplete BIPSS results, presented contradictory results at different time points, and whose tumor was located on both sides.

### 2.4 Surgical Treatment and Prognosis

In total, 417 patients received first-line therapy, transsphenoidal surgery (TSS), of whom 381 patients received endoscopic TSS (eTSS) and 36 patients received microscopic TSS (mTSS); other 5 patients were treated by craniotomy, including subfrontal approach and transorbital approach in consideration of the risk of cerebrospinal fluid (CSF) leakage and scope of operation. The localization, size, shape, texture, and invasiveness of the tumor were recorded during the surgery. Microadenoma and macroadenoma were differentiated on MRI by comparing the maximum diameter with 10 mm. Differently, microadenoma and macroadenoma measured during surgery were based on the size of the excisional part of the tumor. Invasiveness was confirmed by surgeons during the surgery if the cavernous sinus was involved by tumor tissue. All the excised tissues were sent to histopathological analysis and examined by ACTH immunohistochemical staining. Only patients who received TSS were involved in the prognostic analysis. Immediate remission was defined as morning (8 a.m.) serum cortisol concentration lower than 5 μg/dl or 24-h UFC lower than 20 μg/d in a week. Three hundred seventy-seven TSS-treated patients with follow-up of more than 1 year were included in the long-term prognostic analysis.

### 2.5 Statistical Analysis

Statistical analyses were performed with SPSS Statistics 25 software (IBM Corporation, Somers, NY, USA). Continuous variables were compared by independent-samples *t*-test or Wilcoxon test, according to Shapiro-Wilk normality evaluation. Correlation for continuous variables was presented with Spearman correlation coefficient. Categorical variables were analyzed by Pearson’s chi-squared test and Fisher’s exact test.

## 3 Results

### 3.1 Diagnosis

The effectiveness of clinical examinations of CD is listed in **Table 2**. The 24-h UFC was the most sensitive investigation (97 *vs.* 98%), consistent with the previous study (5). Most patients failed to suppress 24-h UFC levels during LDDST (95 *vs.* 97%) and presented positive results on HDDST (93 *vs.* 86%, P = 0.56). The sensitivity of BIPSS test (94 *vs.* 96%) was just behind 24-h UFC and LDDST, again suggesting desmopressin-stimulated BIPSS as an effective alternative to CRH-stimulated BIPSS (6). There was no significant difference in the sensitivity of the above four investigations between pediatric and adult patients. In our study, we also measured the accuracy of pre- and post-stimulation BIPSS test separately. The sensitivity of BIPSS at baseline in pediatric patients was lower than in adults (75 *vs.* 91%, P = 0.054) but increased after desmopressin stimulation (94 *vs.* 95%). However, sampling lateralization could not predict tumor lateralization accurately for both children and adults (45 *vs.* 46%). Further analysis showed that lateralization accuracy of BIPSS for macroadenomas on MRI was not higher than that for microadenomas on MRI (47 *vs.* 40%, P = 0.59), raising the doubt for its effectiveness. MRI identified fewer pituitary adenomas in pediatric patients than with adult patients (87 *vs.* 94%, P = 0.13). The percentage of concordance of microadenomas identified by MRI with surgery was also lower for children (75 *vs.* 89%, P = 0.10). Compared with BIPSS, MRI was more sensitive for localization (pediatric: 79 *vs.* 45%; adults: 72 *vs.* 46%). However, the specificity (33 *vs.* 100%, P = 0.083) and sensitivity (50 *vs.* 32%) of Knosp grading did not achieve satisfying predictive results. After TSS, excised tissues of 27 pediatric and 365 adult patients were confirmed as ACTH-secreting pituitary adenoma.

**Table 2:** Clinical examinations of pediatric and adult-onset CD.

	Pediatric CD patients (n = 30)	Adult CD patients (n = 392)	*P* value
**8 a.m. ACTH > 46 μg/dl**	23 (77)	301 (77)	0.93
**8 a.m. F > 22.3 μg/dl**	23 (77)	289 (74)	0.76
**24-h UFC > 103.5 μg/d**	28 (97)	370 (98)	0.53
**LDDST negative**	21 (95)	271 (97)	0.50
**HDDST positive**	26 (93)	291 (86)	0.56
**BSIPSS sampling**	16 (53)	235 (60)	0.48
**BSIPSS positive**	15 (94)	227 (96)	0.49
**Pre-stimulation BSIPSS positive**	12 (75)	215 (91)	0.054
**Post-stimulation BSIPSS positive**	15 (94)	224 (95)	0.56
**BSIPSS lateralization concordant with surgery**	5 (45)	94 (46)	0.97
**MRI detection**	30 (100)	385 (98)	1
**MRI positive**	26^a^ (87)	361 (94)	0.13
**MRI localization concordant with surgery**	19 (79)	256 (72)	0.44
**Microadenomas on MRI**	14 (74)	237 (81)	0.55
**Microadenomas on MRI concordant with surgery**	12 (75)	245 (89)	0.10
**Invasiveness**	2 (7)	23 (6)	0.70
**MRI Knosp grading ≥ 3**	3 (12)	6 (2)	**0.023**
**Specificity of MRI Knosp grading**	1 (33)	6 (100)	0.083
**Sensitivity of MRI Knosp grading**	1 (50)	6 (32)	1
**Pathology confirmed**	26 (87)	340 (87)	1
**Ki-67 ≥ 3**	7 (25)	101 (27)	0.79

^a^Other four pediatric patients who were MRI negative were all male.

F, serum cortisol.

The bolded values showed the significant results (P < 0.05). No other significant meanings were related.

### 3.2 Manifestations

#### 3.2.1 Gender Distribution

As previously reported (3, 4, 7), there is a significant difference between gender distribution of pediatric and adult-onset CD (P < 0.001). Females dominated the adult CD group (83%), while an equal gender distribution was observed among 30 pediatric patients (50 *vs.* 50%). For children, male patients were older than female patients (14.7 ± 2.8 *vs.* 12.3 ± 4.3, p = 0.075), which is different from adult patients [36.0 (48.3–28.0) *vs.* 39.0 (48.0–31.0), p = 0.23].

#### 3.2.2 Comparison of Clinical Manifestations Between Pediatric and Adult CD Patients

**Figure 1:**
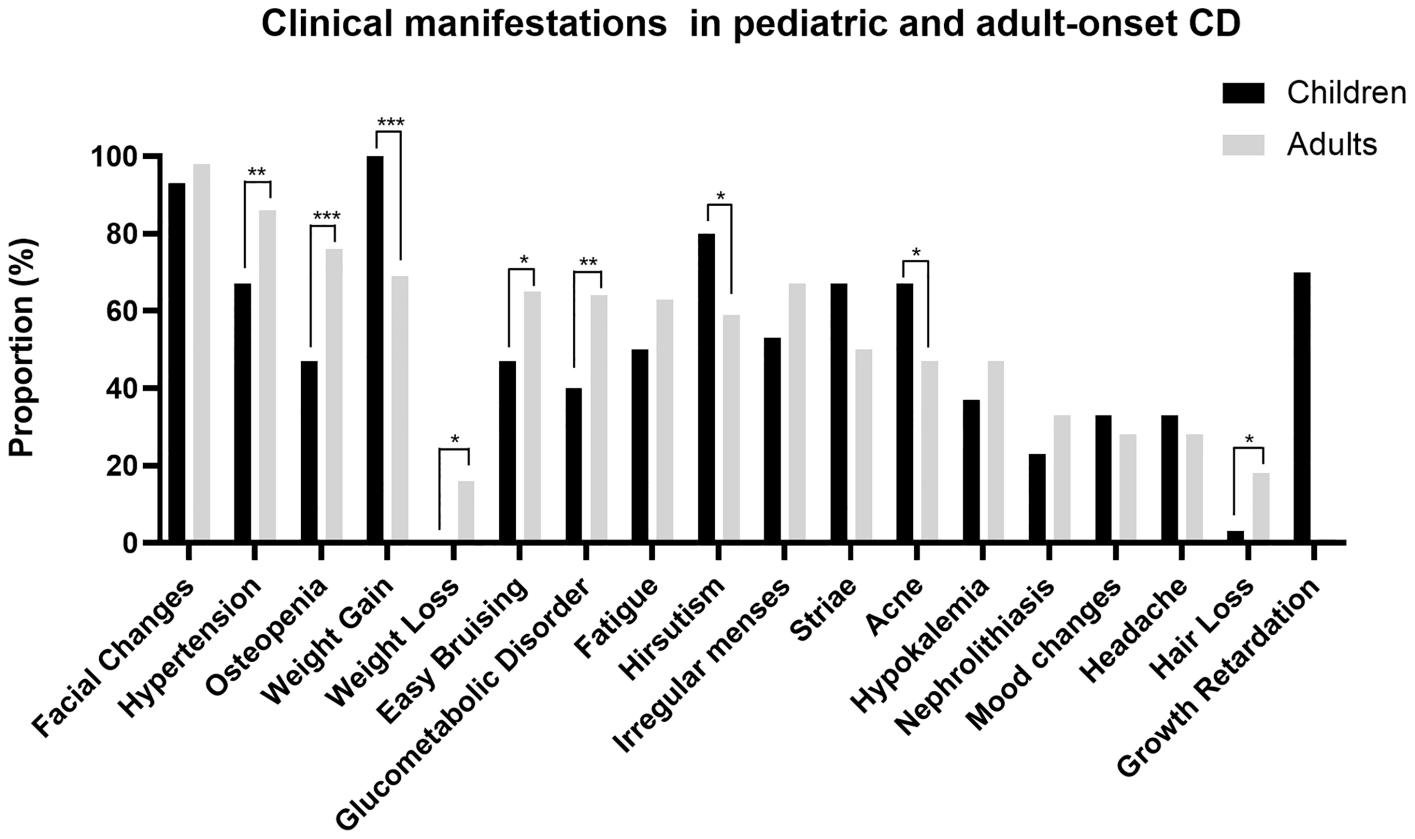
Clinical manifestations in pediatric and adult-onset CD. *P < 0.05, **P < 0.01, ***P < 0.001.

Early recognition of manifestations and symptoms of CD is critical for timely diagnosis and treatment, especially for pediatric patients. In this study, all patients developed more than two typical clinical manifestations shown in **Figure 1** and **Supplementary Table 1**. Similar to previous researches (2, 3, 8), weight gain (100%), facial changes (93%), hirsutism (80%, as a typical sign of virilization), and growth retardation (70%) were the most common manifestations of pediatric CD patients, among which weight gain and hirsutism were more frequently shown compared with adult patients (100 *vs.* 69%, P < 0.001; 80 *vs.* 59%, P = 0.023). Additionally, acne was also more common in pediatric patients (67 *vs.* 47%, P = 0.040). For adults, hypertension were the two most common manifestations compared to pediatric patients (86 *vs.* 67% P = 0.007). Two long-term metabolic-related diseases, osteopenia and glucose intolerance or diabetes, were more common for adults (76 *vs.* 47%, P < 0.001; 64 *vs.* 40%, P = 0.008). Besides, easy bruising and hair loss were also more frequently presented for adults (65 *vs.* 47%, P = 0.047; 18 *vs.* 3%, P = 0.036). Weight loss only occurred in adult patients, with 87% of whom accompanied with glucose intolerance or diabetes.

#### 3.2.3 Comparison of Gender-Related Manifestations Between Pediatric and Adult CD Patients

**Figure 2:**
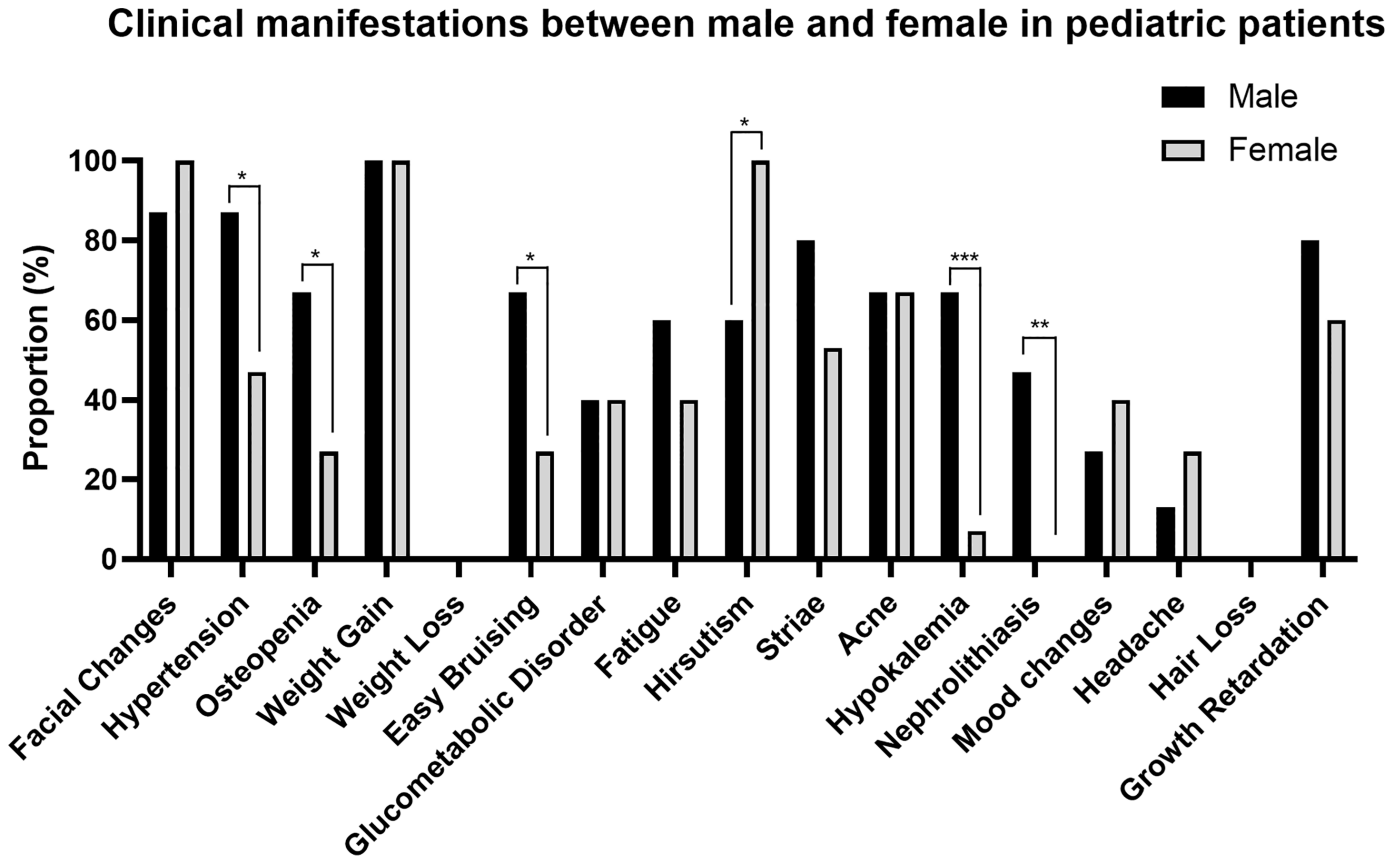
Clinical manifestations between males and females in pediatric patients. *P < 0.05, ** *P* < 0.01, *** *P* < 0.001.

**Figure 3:**
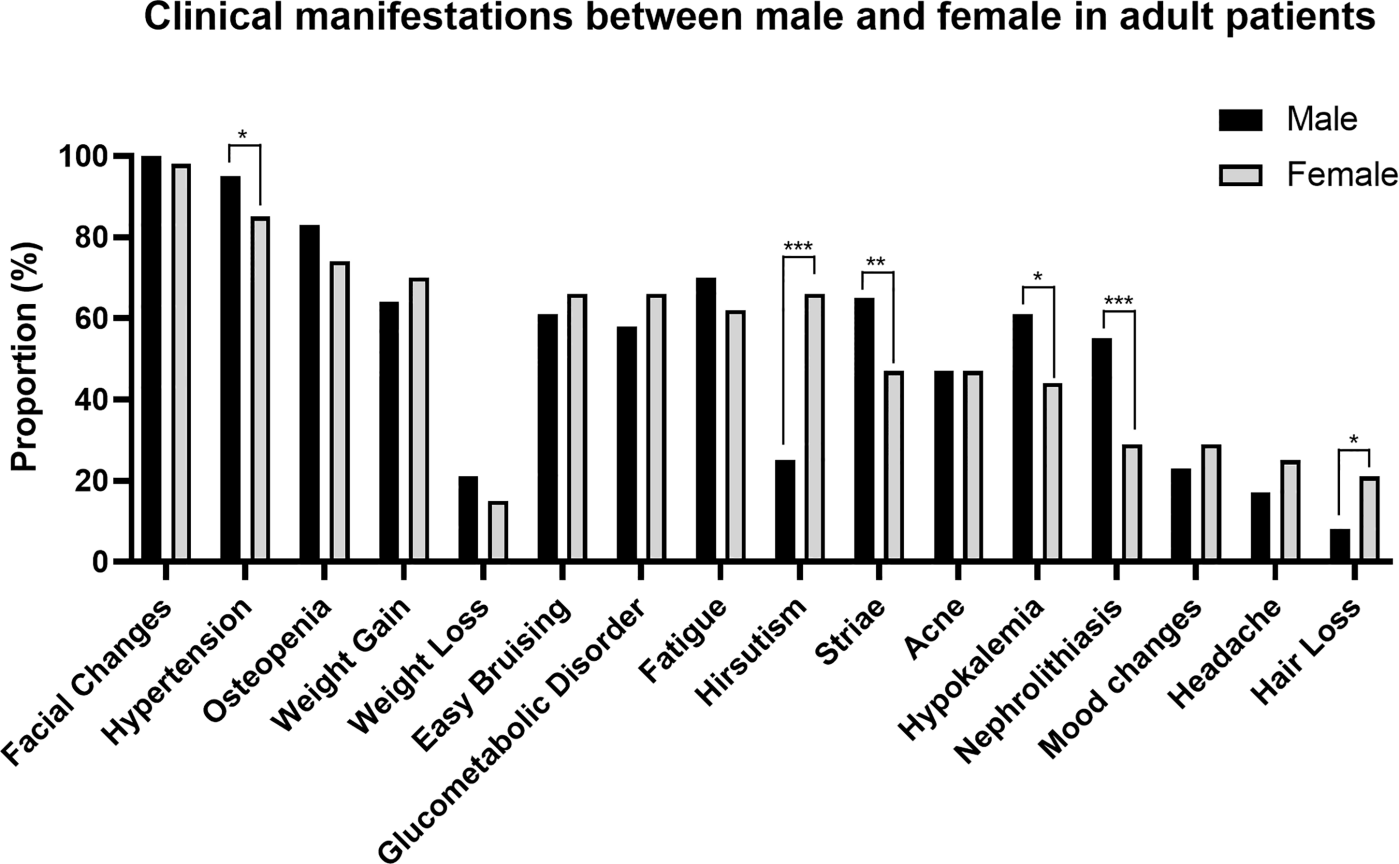
Clinical manifestations between males and females in adult patients. *P < 0.05, **P < 0.01, ***P < 0.001.

Since the gender distribution and the pattern of clinical manifestations are different between pediatric and adult CD patients, we further analyzed the manifestations between male and female in pediatric and adult group respectively **(** **Figures 2**, **3** and **Supplementary Table 2** **)**. Besides the specific presentation of irregular menses for female, two hyperandrogenemia-related manifestations, hirsutism (pediatric: 100 *vs.* 60%, P = 0.017; adult: 66 *vs.* 25%, P < 0.001) and hair loss (adult: 21 *vs.* 8%, P = 0.013), are more common for females. Conversely, the prevalence of nephrolithiasis (pediatric: 47 *vs.* 0%, P = 0.006; adult: 55 *vs.* 29%, P < 0.001), hypokalemia (pediatric: 67 *vs.* 7%, P < 0.001; adult: 61 *vs.* 44%, P = 0.011), hypertension (pediatric: 87 *vs.* 47%, P = 0.020; adult: 95 *vs.* 85%, P = 0.019), easy bruising (pediatric: 67 *vs.* 27%, P = 0.028), osteopenia (pediatric: 67 *vs.* 27%, P = 0.028), and striae (adult: 65 *vs.* 47%, P = 0.006) was greater in male patients, indicating an overall more severe effects and early onset of CD on males.

### 3.3 Laboratory Investigations

#### 3.3.1 Comparison Between Laboratory Investigation Results Between Pediatric and Adult CD Patients

There was no statistically significant difference in plasma ACTH and cortisol at 8 a.m. and 24-h UFC between children and adults, indicating the secretory activity of the PA axis of pediatric patients was not less than adult patients **(** **Supplementary Table 3** **)**. For other laboratory investigations, RBC (P = 0.032) and lymphocyte count (P = 0.005), including B cell (p < 0.001) and CD8^+^ T cell count (P = 0.008), were higher for pediatric patients. Also, a lower CD4^+^/CD8^+^ T cell rate (P = 0.050) and a higher level of ALT (P = 0.001) and AST (P < 0.001) were observed in pediatric patients.

#### 3.3.2 Comparison of Gender-Related Laboratory Investigation Results Between Pediatric and Adult CD Patients

**Figure 4:**
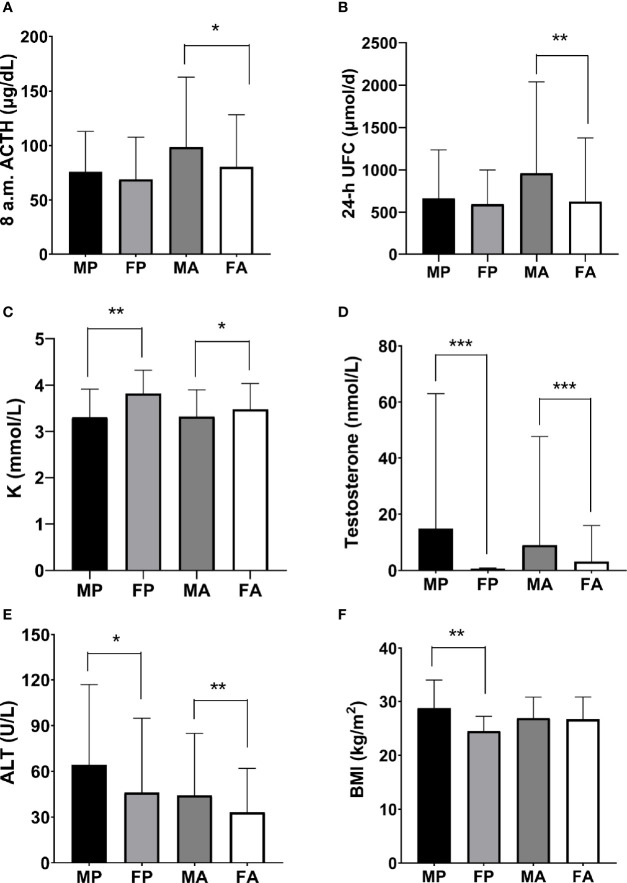
Gender-related laboratory investigation tests in pediatric and adult-onset CD. The parameters of PA activity, **(A)** 8 a.m. ACTH, and **(B)** 24-h UFC, were significantly higher for adult male patients. The levels of **(C)** serum potassium, **(D)** testosterone, and **(E)** ALT were also significantly higher for males in both pediatric and adult patients. **(F)** BMI was significantly higher for male patients in children. MP, male pediatric patients; FP, female pediatric patients; MA, male adult patients; FA, female adult patients. (*P < 0.05, **P < 0.01, ***P < 0.001).

Again, we further analyzed the differences in laboratory investigation results between male and female patients in children and adults, respectively **(** **Figure 4** and **Supplementary Table 4** **)**. Serum potassium level was lower for males in both pediatric (P = 0.010) and adult patients (P = 0.038), which is consistent with the greater prevalence of hypokalemia in male patients mentioned above. For adults, the 24-h UFC (P = 0.003) and 8 a.m. ACTH (P = 0.013) were also higher for male patients, explaining their more severe clinical manifestations. The absolute lymphocyte count (P = 0.070) and CD4^+^ T cell count (P = 0.055) were lower for males in adult patients. Additionally, RBC (P = 0.085; P = 0.085), ALT (P = 0.048; P = 0.003), and testosterone level (P < 0.001; P < 0.001) were higher for male patients. Interestingly, body mass index (BMI) was significantly higher for males than females in pediatric patients (P = 0.012), while there was no such gender-related difference for adult patients.

#### 3.3.3 Correlations Between Pituitary-Adrenal Axis and Laboratory Investigation Results

The 8 a.m. ACTH directly reflects the secretion level of the pituitary, controlling the release of cortisol by the adrenal gland, which is measured by 8 a.m. F level. The 24-h UFC excretion as a sensitive investigation of hypercortisolemia is not affected by changes in cortisol-binding globulin, giving a more stable result (5). As expected, 8 a.m. ACTH level in the macroadenoma group was significantly higher at 87.3 *vs.* 70.9 μg/dl in the microadenoma group (P = 0.019). Likewise, 8 a.m. ACTH/F was also higher for macroadenoma. However, there was no significant difference of 8 a.m. F and 24-h UFC between microadenoma and macroadenoma **(** **Table 3** **)**. Here we use all three investigations as indicators of the PA axis and analyze the correlations with other laboratory tests for male pediatric, female pediatric, male adult, and female adult patients **(** **Table 4** **)**.

**Table 3:** Comparison of investigations of the PA axis between microadenoma and macroadenoma.

	8 a.m. ACTH (μg/dl)	8 a.m. F (μg/dl)	8 a.m. ACTH/F	24-h UFC (μg/d)
**Microadenoma on MRI**	70.9 (86.1–74.3)	26.9 (31.5–26.6)	2.71 (3.81–1.81)	491.8 (806.4–579.3)
**Macroadenoma on MRI**	87.3 (106.1–81.7)	26.0 (30.9–25.1)	3.06 (4.52–2.31)	429.0 (767.5–490.5)
**P value**	**0.019**	0.78	**0.025**	0.61

The bolded values showed the significant results (P < 0.05). No other significant meanings were related.

**Table 4:** Correlation (r_s_) between indicators of the PA axis and other laboratory investigation results.

Patient classification	Indicators of PA axis	WBC	RBC	ALT	AST	K	Lymphocyte	B cell	T cell	CD4^+^ T	CD8^+^ T	CD4^+^ T/CD8^+^ T ratio	BMI	Testosterone
**Male pediatric patients**	8 a.m. ACTH	0.352	−0.158	0.091	−0.143	−0.586^*^	−0.790^**^	−0.663^*^	−0.547	−0.626	−0.182	−0.438	0.070	0.344
	8 a.m. F	0.121	0.174	0.022	0.002	−0.291	−0.600	0.079	−0.697^*^	−0.467	−0.467	−0.212	0.000	−0.014
	24-h UFC	0.538^*^	0.297	−0.059	−0.229	−0.138	−0.527	−0.297	−0.345	−0.285	−0.455	0.006	0.293	−0.215
**Female pediatric patients**	8 a.m. ACTH	0.077	0.088	0.548	−0.186	0.033	−0.500	0.143	−0.500	−0.238	−0.333	−0.048	−0.118	0.564
	8 a.m. F	0.396	0.148	−0.096	0.095	−0.215	0.643	0.143	0.595	0.143	0.571	−0.238	0.129	0.064
	24-h UFC	0.105	0.245	0.375	−0.105	−0.632^*^	−0.179	−0.200	−0.107	−0.643	0.143	−0.786^*^	0.002	0.164
**Male adult patients**	8 a.m. ACTH	0.008	−0.170	0.457^**^	0.243	−0.407^**^	−0.367^*^	0.028	−0.373^*^	−0.392^*^	−0.266	−0.218	0.041	−0.222
	8 a.m. F	0.246	−0.139	0.449^**^	0.213	−0.380^**^	−0.023	0.282	−0.031	−0.099	−0.081	−0.245	0.035	−0.211
	24-h UFC	0.014	−0.206	0.437^**^	0.230	−0.456^**^	−0.073	0.320	−0.117	−0.119	−0.020	−0.219	−0.144	−0.233
**Female adult patients**	8 a.m. ACTH	−0.083	−0.180^**^	0.093	0.080	−0.145^**^	−0.155	0.031	−0.153	−0.211	−0.060	−0.182^*^	−0.094	0.033
	8 a.m. F	0.013	−0.174^**^	0.136^*^	0.051	−0.244^**^	−0.214^**^	−0.017	−0.235^**^	−0.321^**^	−0.148	−0.160^*^	−0.123^*^	0.118^*^
	24-h UFC	0.017	−0.208^**^	0.126^*^	−0.028	−0.189^**^	−0.152	0.027	−0.134	−0.203^*^	−0.005	−0.204^*^	−0.197^**^	0.016

*Correlation is significant at the 0.05 level.

**Correlation is significant at the 0.01 level.

The secretion level on the PA axis was negatively correlated with serum potassium, in concordance with the previous study (9). For male and female adult patients, 8 a.m. ACTH, 8 a.m. F, and 24-h UFC were all negatively correlated with serum potassium. While for male and female pediatric patients, only 8 a.m. ACTH or 24-h UFC was significantly correlated with serum potassium. The excess hormone production on the PA axis caused by CD was also correlated with decreased lymphocytes, especially CD4^+^ T cells. For male adult patients, the negative correlation between 8 a.m. ACTH and lymphocytes, T cells, and CD4^+^ T cells were observed. For female adult patients, lymphocytes, T cells, CD4^+^ T cells, CD4^+^ T/CD8^+^ T ratio were all included. For male pediatric patients, except for lymphocytes and T cells, B cells and CD4^+^ T (with 8 a.m. ACTH: r_s_ = −0.626, P = 0.053) were also correlated with the PA axis. While for female pediatric patients, only CD4^+^ T/CD8^+^ T ratio was correlated with 24-h UFC. Parameters of PA activity were also positively correlated with ALT for adults, and the trend of correlation of 8 a.m. ACTH and 24-h UFC with AST was also detected for male adult patients (r_s_ = 0.243, P = 0.077; r_s_ = 0.230, P = 0.094), suggesting a more severe hepatosis for adult patients. For WBC count, a positive correlation with 24-h UFC was found for male pediatric patients, and the correlation was weaker for male adults (r_s_ = 0.246, P = 0.068). For female adult patients, we observed a negative correlation between PA activity and RBC count, and at the same time, a positive correlation between PA activity and testosterone level. Interestingly, BMI was also found to be negatively correlated with 8 a.m. F and 24-h UFC only for female adults.

### 3.4 Prognosis

We evaluated the accuracy of immediate and long-term remission prediction of the parameters of PA activity, course of the disease, size and invasiveness of tumor, Knosp grading and tumor size predicted on MRI, and whether the patient had received surgery or radiotherapy before **(** **Table 5** **)**. For immediate prognosis, preoperative 8 a.m. ACTH was lower for the remission group (P = 0.004). Patients who had not received surgery or radiotherapy before (P < 0.001), with non-invasive tumor (P < 0.001) or predicted as non-invasive by Knosp grading (P = 0.008), with microadenoma predicted on MRI (P = 0.011), with Ki-67 ≤ 2 (P = 0.074) were more likely to achieve immediate remission. However, due to different levels of invasion, patients with different sizes of tumors resected during surgery showed no significant difference in immediate prognosis. For long-term remission and recurrence, **Table 5** showed no predictor, while interestingly, preoperative 8 a.m. ACTH was higher for long-term remission group (P = 0.064). Overall, the immediate and long-term remission rates were equal for males and females in pediatric and adult patients. The only three recurrent pediatric CD patients were all male.

**Table 5:** Prognosis of patients treated with TSS.

	Immediate remission (%)	Long-term remission (%)	Recurrence (%)
**Excisional microadenoma**	280 (77)	264 (79)	20 (6)
**Excisional macroadenoma**	25 (66)	21 (68)	3 (10)
***P* value**	0.14	0.14	0.43
**Microadenoma on MRI**	203 (81)	45 (80)	4 (7)
**Macroadenoma on MRI**	39 (66)	182 (78)	16 (7)
***P* value**	**0.011**	0.75	1
**Invasive**	8 (0.35)	15 (68)	1 (5)
**Non-invasive**	310 (79)	273 (79)	24 (7)
***P* value**	**<0.001**	0.28	1
**Knosp ≥3**	3 (33)	6 (75)	0 (0)
**Knosp ≤2**	259 (77)	243 (80)	17 (6)
***P* value**	**0.008**	0.67	1
**First-time operation**	283 (79)	247 (79)	21 (7)
**Non-first-time operation**	34 (50)	49 (78)	4 (6)
***P* value**	**<0.001**	0.88	1
**Ki-67 ≥3**	76 (70)	74 (77)	5 (5)
**Ki-67 ≤2**	228 (79)	203 (78)	19 (7)
***P* value**	**0.074**	0.84	0.48

The bolded values showed the significant results (P < 0.05). No other significant meanings were related.

## 4 Discussion

As a rare disease, CD has a substantial impact on the quality of life of patients due to hormonal changes, especially for children in their critical growing stage. Cortisol excess will lead to growth retardation, delayed sexual development, glucose and lipid metabolism disorder, and even emotional lability (2). Thus, the differentiation and diagnosis of CD are very challenging and essential. Several studies paid attention to pediatric CD patients, and Storr focused on comparing diagnostic and clinical features between pediatric and adult-onset CD. To our knowledge, there is no reported systematic comparison on the accuracy of diagnosis, clinical features, laboratory investigations, and prognosis between pediatric and adult CD, and at the same time, considering the impact of CD on different gender.

### 4.1 Accuracy and Clinical Value of Different Diagnostic Methods

In our cohort, 24-h UFC was the most accurate investigation for hypercortisolemia for both pediatric and adult patients. BIPSS with desmopressin stimulation yielded comparable accuracy to 24-h UFC measurement. Though the sampling rate was relatively low (**Table 2**) as an invasive examination, BIPSS could confirm 100% 24-h UFC < 103.5 μg/d, 96% MRI negative, 97% HDDST negative cases as CD. It is beneficial as an accessory examination, especially for its specificity and high accuracy in distinguishing CD and EAS (6, 10). We found that the sensitivity of BIPSS at baseline was lower than after stimulation, especially for children, indicating that the stimulation of desmopressin is very necessary to increase the sensitivity of BIPSS in these cases, verifying that desmopressin is an efficient alternative to CRH (6). Similarly, such an increase was also observed in the previous study (10). Chen reported that the optimal cutoff of BIPSS was 1.4 before stimulation and 2.8 after stimulation based on a cohort including 226 CD and 24 ectopic adrenocorticotropin syndrome (EAS) patients in the endocrinology department also in Peking Union Medical College Hospital (11). Using the new criteria, the sensitivity of baseline BIPSS for children in our cohort reached 94%. The specificity and accuracy for differentiation diagnosis of this new criteria still need to be confirmed for pediatric patients. For lateralization, as several studies indicated, the concordance with surgery was disappointing (10, 12, 13). Since the asymmetric parasellar venous drainage pattern and asymmetric bilateral catheter positioning could all diminish the predictive value, we do not recommend using BIPSS to predict lateralization of pituitary adenoma (14).

Localization on MRI was more precise, yet the predictive value of tumor invasiveness on MRI still needed improvement. Also, some microadenomas could not be exactly seen on MRI scan. Similar to Storr’s research, a lower percentage of adenomas were confirmed on MRI in children, and the concordance of tumor size on MRI with surgery was also lower for them. However, different from previous discoveries, there was no difference in the percentage of microadenoma on MRI between children and adults (3). As for the relationship between tumor size and CD, some previous studies with smaller sample sizes reached different conclusions. Mathioudakis and Kakade found that macroadenomas had lower serum cortisol in comparison with microadenomas (9, 15), while Witek discovered higher ACTH secretion by macroadenomas (16). But a higher ACTH/serum cortisol ratio for macroadenomas seemed to be the same result. Similar to Witek, in our study, only 8 a.m. ACTH level and 8 a.m. ACTH/F were higher in the macroadenoma group, suggesting that macroadenoma was not necessarily correlated with a higher response in the lower PA axis, and thus the severity of CD.

Except for the preoperative ACTH level, invasiveness, and Ki-67, we mainly depend on MRI to provide clues, including the size and Knosp grading (16), to predict immediate prognosis, illustrating that MRI indeed contains a lot of information. But still, the long-term prognosis is hard to predict by existing clinical examinations, calling for the use of radiomics. Besides, the development of machine learning also helped to optimize the algorithm of immediate and delayed remission prediction, as well as automatically identify CD patients by recognizing anomalies in facial images (17, 18).

### 4.2 CD’s Impact on Laboratory Parameters and Clinical Manifestations for Pediatric and Adult Patients

For laboratory investigations, we observed a higher level of RBC count, lymphocyte count including B cell and CD8^+^ T cell, ALT, and AST for pediatric patients. ALT and AST are not only distributed in the liver but also stored in the skeletal muscle, indicating the level of growth and development (19, 20). So it was not surprising to see a higher range of ALT and AST for children. Only for adult patients did we observe a positive correlation of the secretion along the PA axis with ALT and AST, implying the relation of CD and liver dysfunction for adults. Blood cell counts vary with age. The absolute numbers of T and B cells for children are higher and gradually decrease to adult levels at about 13 years old (21), while RBC count gradually increases with age to a maintenance level (22). So it seems that the higher RBC count and lower CD4^+^/CD8^+^ T cell for pediatric patients could not be partially explained by physiological changes. In addition, since the absence of the control group of healthy children, these parameters still need to be carefully interpreted. For other investigations, including the activity of the PA axis, because there was no significant difference between children and adult groups, the impact of CD on growing children might be more profound and lasting, calling for special attention. Comparing the clinical features, children tended to have a more rapid onset and obvious manifestations, including weight gain and growth retardation, driving them to seek treatments. Similar to Storr’s research, we found that children were more sensitive to the change of sex hormone, particularly androgen, presented as a higher prevalence of hirsutism and acne. The suppressive effect of hypercortisolemia on the pituitary-gonadal (PG) axis have been widely reported (23). At the same time, excess circulating ACTH will stimulate androgen production in the adrenal cortex (24). Since the development of children’s gonads is not mature enough, the suppressed gonadal androgen can be compensated by the excess androgen, contributing to the virilization of pediatric CD patients. But the suppression of PG function on children’s immature gonads can be more alarming because of its impact on delayed sexual development (2). In **Supplementary Table 1**, we included all female patients. For patients between menophania and menopause, all pediatric patients developed irregular menses, while the ratio for adults was 78%, indicating a strong impact on children. Furthermore, hypertension, osteopenia, glucose intolerance, fatigue, bruising, striae, mood change, and hypokalemia were also prevalent for pediatric patients and would probably further progress and develop into lifelong complications. Therefore, accurate diagnosis and early treatment are crucial to pediatric patients’ normalization of metabolism and development both physically and mentally (7, 25, 26).

For adult patients, the prevalence of chronic diseases and metabolic syndromes was higher, including hypertension, osteoporosis, muscle weakness, fatigue, impaired glucose metabolism that explains weight loss, hypokalemia, nephrolithiasis, and androgen-related alopecia. Some of the manifestations, particularly hypertension and abnormal glucose metabolism, need special attention because they are related to a higher risk of cardiovascular and cerebrovascular diseases, which are the most common causes of death (27). Note that age is also an important influence factor for many of the chronic diseases mentioned above, so the long-term management and the differentiation of CD with these kinds of diseases are critical.

### 4.3 CD’s Impact on Laboratory Parameters and Clinical Manifestations for Male and Female Patients

Gender distribution in pediatric CD patients was analyzed in many studies, some of which reported male predominance. Same as a large series of 102 patients from the NIH, our study showed an equal gender distribution (7). So far, the reason for the higher male proportion in pediatric patients compared with adults is not clear. By comparing the manifestations, laboratory investigations, and prognosis between different gender in the pediatric and adult groups, we speculated that male patients develop more severe symptoms at a younger age. Comparing the PA activity, we found that 8 a.m. ACTH and 24-h UFC were significantly higher for males in the adult group despite the prevalence of microadenomas on MRI was similar for different gender, indicating a more pronounced secretory activity. Whereas for children, male patients did not present higher PA activity, but excess cortisol seemed to have a more profound influence on their growing bodies under different levels of sex hormones, resulting in more severe clinical manifestations.

Correlation analysis showed that the level of serum potassium and the number of lymphocytes, especially CD4^+^ T cells, were negatively correlated with parameters of PA secretion level for both genders in children and adults. Cortisol can bind to the mineralocorticoid receptor on the collecting tubule and promote the excretion of potassium and reabsorption of sodium, contributing to hypokalemia. Also, a previous study has reported the negative correlation between plasma potassium and cortisol levels (28). As for the immune regulation of CD, lymphopenia, reduced CD4^+^ T cell count, and a reduced ratio of CD4^+^/CD8^+^ T cells have been reported in CD patients (8). In contrast, only the absolute lymphocyte count has been demonstrated to be negatively correlated with serum cortisol and 24-h UFC level (29, 30). The mechanism of lymphopenia has been partially revealed by an *in vitro* study that T cells were sensitive to GC-induced apoptosis, especially for CD8^+^ T cells (31). However, this laboratory finding could not explain the reduced ratio of CD4^+^/CD8^+^ T cells and other cell types linked to CD.

In the present study, we found that hypokalemia was more prevalent for males in both pediatric and adult patients, and the absolute serum potassium level was also lower for males. The significant difference in the degree of hypokalemia between different genders has not been reported and explained. For the immune system, we found that lymphocytes and CD4^+^ T cells were lower for adult male patients, while the number of lymphocytes, B cells, T cells, and CD4^+^ T cells also seemed to be lower for pediatric male patients. The overall more profound effect of immune suppression for male patients implies the higher possibility of infections, which is related to a higher risk of mortality (8, 29). Another gender-related parameter was BMI, which was significantly higher for males in pediatric patients, possibly resulting in the early care-seeking behavior for male children. The increased adipose tissue will lead to metabolic changes, including leptin, resistin, and pro-inflammatory factors, leading to insulin resistance and other cardiovascular and cerebrovascular diseases (8). Considering other clinical manifestations, we found that female-prevalent symptoms were all complications of hyperandrogenism, consisting of alopecia and hirsutism. That’s because, compared with male adults, the PG function only accounts for half of the total androgen. Therefore, the excess ACTH secretion could have an opposite impact on serum testosterone levels for male and female CD patients. For other manifestations, the prevalence of nephrolithiasis and hypertension was more remarkable for male patients. Osteoporosis and ecchymosis were more common for males in the pediatric group, indicating the early onset of these symptoms for males. Furthermore, striae presence was also higher for males in adult patients.

In summary, the secretory activity of the PA axis was higher for males, contributing to the more remarkable alteration of long-term metabolism and immune system, presenting as more severe and earlier onset of a series of symptoms. Besides, the pituitary adenoma was less confirmed by MRI and recurred more frequently after TSS surgery for males in the pediatric group, supporting the more dangerous nature of pituitary adenoma for male patients. But the mechanism behind the gender-related difference in the characteristics of CD is still unknown. The results of our study were similar to previous literature, which concluded that male CD patients showed more severe clinical presentation, higher ACTH and cortisol levels (32). However, our study recruited more patients and compared the gender-related differences in the pediatric and adult group, achieving more significant results supporting the more serious CD in males.

### 4.4 Limitations and Strengths

Our study has several limitations. First, we did not recruit a control group of matched healthy children and adults, so we could not get a clearer view of the degree of CD’s impact on different kinds of patients. Second, we did not include patients showing hypercortisolemia caused by other diseases such as EAS. Therefore, we could not measure the specificity of some diagnostic methods. Third, we screened patients with follow-up visits longer than 1 year for the long-term prognosis part. However, the time was still not long enough to observe the recovery of chronic and metabolic complications of CD. Fourth, because this is a retrospective study, we could not avoid missing values, especially for the results of laboratory investigations that were recorded postoperatively and during follow-up visits. Without the comparison between pre- and post-operation laboratory tests, we could not exclude the heterogeneity between different patients and evaluate the change after TSS surgery. Last, although we included a large number of 422 patients in total, the number of pediatric patients was relatively insufficient due to lower morbidity, limiting the accuracy and significance of the results related to children.

Our study contains the largest single-center cohort including both pediatric and adult CD patients, contributing high-value and reliable clinical evidence to the preclinical and clinical field of CD-related researches. Compared with other studies that compared the impact of CD on different groups of patients, we are the first to consider both factors of age and gender. Additionally, all patients received pituitary adenoma excision by only one experienced surgeon, controlling the impact of operational heterogeneity on prognosis. We successfully observed a more severe and probably an earlier-onset pattern of CD for male patients, reminding the clinicians during their clinical practices, and at the same time, promoting deeper researches into the mechanism of pathogenesis of CD for different patient groups.

To conclude, in this paper, we present a retrospective study comparing the diagnosis, symptoms, preoperative laboratory parameters, and prognosis of CD for pediatric and adult patients. To explore the reason for the higher male proportion in pediatric CD patients, we further analyzed the impact of CD on different genders in children and adults, observing a more severe and probably an earlier-onset pattern of CD for male patients.

## Data Availability Statement

Dataset is accessible through contact with the corresponding author. Requests to access these datasets should be directed to XZ, 1608596426@qq.com.

## Ethics Statement

The studies involving human participants were reviewed and approved by the Institutional Review Board of Peking Union Medical College Hospital. Written informed consent from the participants’ legal guardian/next of kin was not required to participate in this study in accordance with the national legislation and the institutional requirements.

## Author Contributions

XZ: Conceptualization, methodology, formal analysis, investigation, data curation, writing—original draft, visualization. HW: Methodology, writing—original draft. WZ: Conceptualization, methodology, data curation. SF: Methodology, data curation, resources. YL: Investigation. SL: Investigation. XB: Data curation, resources. LL: Data curation, resources. HZ: Data curation, resources. MF: Conceptualization, methodology, validation, writing—review and editing, supervision, project administration. RW: Supervision, project administration. All authors contributed to the article and approved the submitted version.

## Funding

This work was supported by the reforming programme on education for graduates in PUMC (10023201900107).

## Conflict of Interest

The authors declare that the research was conducted in the absence of any commercial or financial relationships that could be construed as a potential conflict of interest.

## Publisher’s Note

All claims expressed in this article are solely those of the authors and do not necessarily represent those of their affiliated organizations, or those of the publisher, the editors and the reviewers. Any product that may be evaluated in this article, or claim that may be made by its manufacturer, is not guaranteed or endorsed by the publisher.
