# Supplementary material for: Diagnosis, Manifestations, Laboratory Investigations, and Prognosis in Pediatric and Adult Cushing’s Disease in a Large Center in China
Source: Front Endocrinol (Lausanne). 2021 Nov 19;12:749246. doi: 10.3389/fendo.2021.749246 (PMC8640923; doi:10.3389/fendo.2021.749246)
Supplement: Supplementary file 1 [file Table_1.docx]

**Supplementary Table 1: Clinical manifestations and ages at diagnosis in pediatric and adult-onset CD**

| **Clinical manifestations** | **Pediatric CD patients (n=30)** | | **Adult CD patients (n=392)** | **P value** |
| --- | --- | --- | --- | --- |
|  | **n (%)** | **Mean age ± S.D. (years)** | **n (%)** |  |
| **Facial changes** | 28 (93) | 13.3±3.8 | 386 (98) | 0.105 |
| **Hypertension** | 20 (67) | 14.2±3.5 | 339 (86) | **0.007** |
| **Osteopenia** | 14 (47) | 14.5±3.0 | 297 (76) | **<0.001** |
| **Weight gain** | 30 (100) | 13.5±3.7 | 271 (69) | **<0.001** |
| **Weight loss** | 0 (0) |  | 62 (16) | **0.013** |
| **Easy bruising** | 14 (47) | 13.3±3.6 | 254 (65) | **0.047** |
| **Glucometabolic disorder** | 12 (40) | 13.6±3.3 | 252 (64) | **0.008** |
| **Fatigue** | 15 (50) | 14.5±3.1 | 263 (63) | 0.15 |
| **Hirsutism** | 24 (80) | 13.1±4.0 | 231 (59) | **0.023** |
| **Irregular menses (female)^a^** | 8 (53) | 15.6±1.7 | 217 (67) | 0.29 |
| **Striae** | 20 (67) | 15.0±3.8 | 195 (50) | 0.074 |
| **Acne** | 20 (67) | 14.2±3.5 | 185 (47) | **0.040** |
| **Hypokalemia** | 11 (37) | 14.6±3.2 | 184 (47) | 0.28 |
| **Nephrolithiasis** | 7 (23) | 15.1±1.6 | 131 (33) | 0.26 |
| **Mood changes** | 10 (33) | 12.4±4.3 | 110 (28) | 0.54 |
| **Headache** | 6 (20) | 13.7±2.4 | 94 (24) | 0.62 |
| **Hair loss** | 1 (3) | (age 17) | 72 (18) | **0.036** |
| **Growth retardation** | 21 (70) | 12.9±3.5 | 2 (1) |  |

a: All female patients were included. 7 children were before menophania and 47 adults were after menopause.

**Supplementary Table 2: Gender-related manifestations in pediatric and adult-onset CD**

| **Clinical manifestations** | **Pediatric CD patients (n=30)** | | | **Adult CD patients (n=392)** | | |
| --- | --- | --- | --- | --- | --- | --- |
|  | **n in male (%)** | **n in female (%)** | **P value** | **n in male (%)** | **n in female (%)** | **P value** |
| **Facial changes** | 13 (87) | 15 (100) | 0.48 | 66 (100) | 320 (98) | 0.60 |
| **Hypertension** | 13 (87) | 7 (47) | **0.020** | 63 (95) | 276 (85) | **0.019** |
| **Osteopenia** | 10 (67) | 4 (27) | **0.028** | 55 (83) | 242 (74) | 0.12 |
| **Weight gain** | 15 (100) | 15 (100) | 1 | 42 (64) | 229 (70) | 0.29 |
| **Weight loss** | 0 (0) | 0 (0) | 1 | 14 (21) | 48 (15) | 0.17 |
| **Easy bruising** | 10 (67) | 4 (27) | **0.028** | 40 (61) | 214 (66) | 0.43 |
| **Glucometabolic disorder** | 6 (40) | 6 (40) | 1 | 38 (58) | 214 (66) | 0.21 |
| **Fatigue** | 9 (60) | 6 (40) | 0.27 | 46 (70) | 202 (62) | 0.23 |
| **Hirsutism** | 9 (60) | 15 (100) | **0.017** | 17 (25) | 214 (66) | **<0.001** |
| **Striae** | 12 (80) | 8 (53) | 0.12 | 43 (65) | 152 (47) | **0.006** |
| **Acne** | 10 (67) | 10 (67) | 1 | 31 (47) | 154 (47) | 0.97 |
| **Hypokalemia** | 10 (67) | 1 (7) | **<0.001** | 40 (61) | 155 (44) | **0.011** |
| **Nephrolithiasis** | 7 (47) | 0 (0) | **0.006** | 36 (55) | 95 (29) | **<0.001** |
| **Mood changes** | 4 (27) | 6 (40) | 0.44 | 15 (23) | 95 (29) | 0.29 |
| **Headache** | 2 (13) | 4 (27) | 0.65 | 11 (17) | 83 (25) | 0.13 |
| **Hair loss** | 0 (0) | 0 (0) | 1 | 5 (8) | 67 (21) | **0.013** |
| **Growth retardation** | 12 (80) | 6 (60) | 0.43 |  |  |  |

**Supplementary Table 3: Laboratory investigation results in pediatric and adult-onset CD**

| **Laboratory investigations** | **Pediatric CD patients (n=30)** | **Adult CD patients (n=392)** | **P value** |
| --- | --- | --- | --- |
| **8 a.m. ACTH (μg/dL)** | 64.4 (93.9-45.3) | 72.6 (107.0-49.3) | 0.30 |
| **8 a.m. F (μg/dL)** | 27.6 (33.3-23.1) | 26.9 (33.0-22.1) | 0.60 |
| **24-h UFC (μg/dL)** | 492.6 (858.1-275.6) | 480.5 (781.3-290.0) | 0.92 |
| **WBC count (10^3^/μL)** | 8.3 (9.7-6.7) | 8.4 (10.2-6.9) | 0.96 |
| **RBC count (10^6^/μL)** | 4.7 (5.0-4.3) | 4.5 (4.7-4.2) | **0.032** |
| **ALT (U/L)** | 40 (58-30) | 27.5 (39.8-20.0) | **0.001** |
| **AST (U/L)** | 23.5 (30.3-20.0) | 19.0 (24.0-16.0) | **<0.001** |
| **K (mmol/L)** | 3.7 (3.9-3.1) | 3.5 (3.9-3.1) | 0.26 |
| **Lymphocyte count (10^3^/μl)** | 1768.0 (2163.8-1389.0) | 1331.0 (1785.0-1019.3) | **0.005** |
| **B cell count (10^3^/μL)** | 278.0 (443.0-196.3) | 123.0 (176.3-75.0) | **0.000** |
| **T cell count (10^3^/μL)** | 1162.5 (1671.3-1015.8) | 990.0 (1336.0-762.0) | **0.049** |
| **CD4^+^ T cell count (10^3^/μL)** | 522.0 (619.8-358.3) | 485.5 (700.3-348.3) | 0.91 |
| **CD8^+^ T cell count (10^3^/μL)** | 544.5 (890.5-452.3) | 407.0 (593.0-322.0) | **0.008** |
| **CD4^+^/ CD8^+^ T cell** | 0.95 (1.26-0.58) | 1.15 (1.54-0.80) | **0.050** |
| **BMI (kg/m^2^)** | 26.8 (28.4-23.9) | 25.9 (28.5-24.3) | 0.868 |
| **Testosterone (nmol/L)** | 0.79 (1.21-0.50) | 0.92 (1.34-0.64) | 0.272 |

F: serum cortisol; WBC: White blood cell; RBC: Red blood cell; ALT: alanine aminotransferase; AST: aspartate transaminase; K: serum potassium; BMI: Body mass index

**Supplementary Table 4: Gender-related laboratory investigation tests in pediatric and adult-onset CD**

| **Laboratory investigations** | **Pediatric CD patients (n=30)** | | | | **Adult CD patients (n=392)** | | | |
| --- | --- | --- | --- | --- | --- | --- | --- | --- |
|  | **male** | **female** | **P value** | **male** | | **female** | **P value** |  |
| **8 a.m. ACTH (μg/dL)** | 64.7 (103.0-50.6) | 59.9 (93.5-33.1) | 0.49 | 86.6 (123.3-59.4) | | 69.1 (105.0-47.1) | **0.013** |  |
| **8 a.m. F (μg/dL)** | 27.2 (41.9-23.6) | 28.1 (33.3-20.6) | 0.57 | 28.4 (34.4-22.6) | | 27.7 (32.2-22.0) | 0.315 |  |
| **24-h UFC (μg/d)** | 492.6 (856.7-262.8) | 486.1 (888.8-277.8) | 0.95 | 590.5 (1281.2-373.1) | | 467.6 (741.6-273.7) | **0.003** |  |
| **WBC count (10^3^/μL)** | 8.9 (11.6-7.1) | 7.9 (9.1-6.7) | 0.19 | 8.5 (10.9-7.3) | | 8.2 (10.2-6.8) | 0.071 |  |
| **RBC count (10^6^/μL)** | 4.8 (5.1-4.6) | 4.5 (4.9-4.1) | 0.085 | 4.6 (4.8-4.2) | | 4.4 (4.7-4.2) | 0.085 |  |
| **ALT (U/L)** | 50.5 (71.8-37.5) | 33.0 (44.0-20.0) | **0.048** | 32.0 (49.3-24.0) | | 27.0 (38.0-19.0) | **0.003** |  |
| **AST (U/L)** | 28.0 (35.3-20.8) | 22.0 (26.5-19.3) | 0.118 | 20.0 (24.0-16.0) | | 19.0 (23.3-16.0) | 0.52 |  |
| **K (mmol/L)** | 3.3 (3.6-3.0) | 3.8 (4.2-3.7) | **0.010** | 3.3 (3.8-3.0) | | 3.5 (3.9-3.1) | **0.038** |  |
| **Lymphocyte count (10^3^/μL)** | 1724.5 (1952.0-1285.3) | 1920.5 (2655.0-1572.0) | 0.36 | 1080.0 (1681.0-942.5) | | 1386.0 (1795.5-1055.5) | 0.070 |  |
| **B cell count (10^3^/μL)** | 233.5 (389.0-172.5) | 378.5 (498.8-211.0) | 0.31 | 124.5 (164.5-61.3) | | 121.5 (177.0-77.3) | 0.37 |  |
| **T cell count (10^3^/μL)** | 1096.0 (1471.8-869.5) | 1278.5 (1955.3-1124.5) | 0.17 | 854.0 (1274.8-653.3) | | 1015.0 (1367.5-784.5) | 0.17 |  |
| **CD4^+^ T cell count (10^3^/μL)** | 446.5 (688.0-309.5) | 551.0 (619.8-409.5) | 0.41 | 377.5 (644.8-267.5) | | 509.0 (713.0-361.5) | 0.055 |  |
| **CD8^+^ T cell count (10^3^/μL)** | 544.5 (690.8-470.3) | 566.5 (1009.8-336.5) | 0.97 | 357.5 (511.0-299.5) | | 421.0 (601.5-323.0) | 0.11 |  |
| **CD4^+^ T / CD8^+^ T ratio** | 0.83 (1.11-0.55) | 1.13 (1.33-0.67) | 0.32 | 1.10 (1.41-0.77) | | 1.16 (1.55-0.81) | 0.61 |  |
| **BMI (kg/m^2^)** | 28.3 (32.9-26.8) | 25.0 (26.8-21.2) | **0.007** | 25.8 (27.9-24.8) | | 26.0 (28.6-24.1) | 0.93 |  |
| **Testosterone (nmol/L)** | 1.15 (2.73-0.94) | 0.61 (0.76-0.36) | **<0.001** | 2.04 (2.7-1.5) | | 0.67 (0.95-0.48) | **<0.001** |  |
